# Effect and Mechanism of Mitomycin C Combined with Recombinant Adeno-Associated Virus Type II against Glioma

**DOI:** 10.3390/ijms15010001

**Published:** 2013-12-19

**Authors:** Hong Ma, Yunjia Zhang, Hailong Wang, Chuanhui Han, Runhong Lei, Lei Zhang, Zuye Yang, Ling Rao, Hong Qing, Jim Xiang, Yulin Deng

**Affiliations:** School of Life Science, Beijing Institute of Technology, Haidian District, Beijing 100081, China; E-Mails: 1122334@bit.edu.cn (Y.Z.); whailong1983@126.com (H.W.); mah_cindy@sina.com (C.H.); leirh0581@gmail.com (R.L.); zhaon19900709@163.com (L.Z.); leirh0581@mails.jlu.edu.cn (Z.Y.); raolingok@126.com (L.R.); hqing@bit.edu.cn (H.Q.); jim.xiang@saskcancer.ca (J.X.)

**Keywords:** recombinant adeno-associated virus II, mitomycin C, glioma

## Abstract

The effect of chemotherapy drug Mitomycin C (MMC) in combination with recombinant adeno-associated virus II (rAAV2) in cancer therapy was investigated, and the mechanism of MMC affecting rAAV2’s bioactivity was also studied. The combination effect was evaluated by the level of GFP and TNF expression in a human glioma cell line, and the mechanism of MMC effects on rAAV mediated gene expression was investigated by AAV transduction related signal molecules. C57 and BALB/c nude mice were injected with rAAV-*EGFP* or rAAV-*TNF* alone, or mixed with MMC, to evaluate the effect of MMC on AAV-mediated gene expression and tumor suppression. MMC was shown to improve the infection activity of rAAV2 both *in vitro* and *in vivo*. Enhancement was found to be independent of initial rAAV2 receptor binding stage or subsequent second-strand synthesis of target DNA, but was related to cell cycle retardation followed by blocked genome degradation. *In vivo* injection of MMC combined with rAAV2 into the tumors of the animals resulted in significant suppression of tumor growth. It was thus demonstrated for the first time that MMC could enhance the expression level of the target gene mediated by rAAV2. The combination of rAAV2 and MMC may be a promising strategy in cancer therapy.

## 1. Introduction

Adeno-associated virus (AAV) is a small non-enveloped virus and 11 AAV serotypes have been reported. AAV2 has become a widely used serotype [1,2]. AAV can infect both dividing and non-diving cells, persist in human bodies and show minimal immunogenic effect and no pathogenicity at all in preclinical data from murine models. These attractive characteristics make AAV a very competitive candidate for creating viral vectors for gene therapy [3]. Recombinant adeno-associated virus type II (rAAV2) consists of promoter elements and genes which are flanked by the inverted terminal repeats (ITRs). rAAV2 could prolong transgene expression and is used for clinical trials and others, such as treatment of Canavan disease, muscular dystrophy and tumors [3].

RAAV infects cells through several stages, from binding to its primary receptor [4] on the cell surface followed by endocytosis through co-receptors [5–7] to migration to the nucleus through several steps, including vesicular trafficking, endosomal escape and nuclear transportion and the final post-nuclear stages including viral uncoating, second-strand synthesis and circularization [8]. Due to the complexity of rAAV’s infection pathway, some stages can be biologic barriers to affect gene delivery and expression, like receptor binding stage [9,10] and genome conversion from single strand to double strand [11,12]. Consequently, combining rAAV mediated gene therapy with other therapies has been considered as a preferred method. Both gamma ray and UV light can enhance transgene expression and transduction efficiency [11,12]. Moreover, tritiated thymidine [13], some antitumor drugs [14–17] as well as some DNA-damaging agents have also been considered to increase the efficiency of rAAV transduction in various cells [18,19]. Mitomycin C (MMC) is a potent DNA crosslinker that alkylates double strand DNA [20] and has been widely used as a chemotherapeutic agent [21]. To date, the role of MMC in the enhancement of AAV-mediated gene expression has not been fully studied.

In this study, we focus on the effect and mechanism of MMC on the efficiency of gene delivery mediated by rAAV2. It is demonstrated that administration of MMC can enhance rAAV-mediated transgene expression, but facilitation of transduction efficiency was not observed. Moreover, MMC cannot modify receptor expression during the course of rAAV infection, but can affect cell genome stability. Cell cycle arrest in S phase may contribute to genome DNA delaying, thereafter causing higher level of gene expression. The same enhancing effect has also been observed in an experimental animal model. These findings make the strategy of combining MMC with AAV2-mediated gene therapy against human glioma promising.

## 2. Results and Discussion

### 2.1. The Sensitivity of U251 Cell Line to MMC and rAAV-TNF *in Vitro*

**Figure 1.**
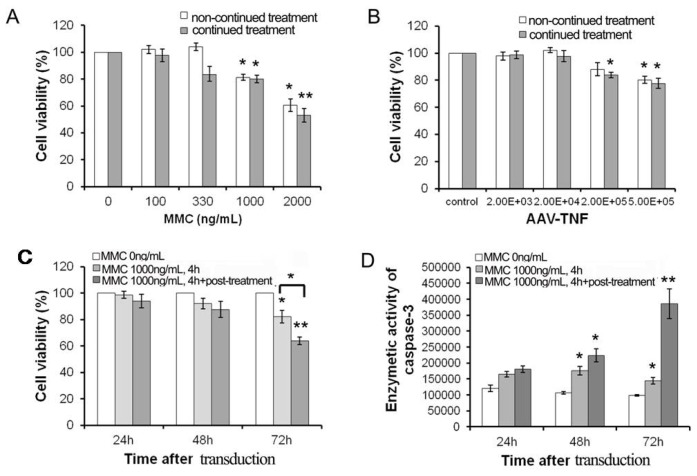
Characteristics of human glioma cell line U251 for MMC and rAAV treatment. (**A**) the cell viability at 72 h after non-continued (pretreatment for 4 h) or continued (pretreatment for 4 h and post-treatment for 72 h) treatment with different dosages of MMC, and cell treated with vehicle solution as control; (**B**) the cell viability at 72 h after non-continued (pretreatment for 4 h) or continued (pretreatment for 4 h and post-treatment for 72 h) infection with different dosages AAV-*TNF*, and cell infected with AAV-*EGFP* as control; (**C**) the cell viability after AAV-*TNF* infection with MMC (1000 ng/mL) non-continued (pretreatment for 4 h) or continued (pretreatment for 4 h and post-treatment for different time) co-treatment, and cell only infected with AAV-*TNF* as control; and (**D**) the assay of enzymatic activity of caspase-3 after AAV-*TNF* infection with MMC (1000 ng/mL) non-continued (pretreatment for 4 h) or continued (pretreatment for 4 h and post-treatment for different time) co-treatment, and cell only infected with AAV-*TNF* as control (* *p* < 0.05, ** *p* < 0.01).

The sensitivity of the U251 cell line to MMC was first examined. As shown in Figure 1A, only 50% of U251 cells survived for the continued treatment at the dose of 2000 ng/mL MMC, while about 80% survived at 1000 ng/mL for either non-continued or continued treatment. Therefore the resistant dosage of MMC can be confirmed as 1000 ng/mL. An effective dosage for rAAV-*TNF* effect on U251 cells was chosen according to an introduction with doses of (2 × 10^3^)−(5 × 10^5^) Gps/cell. The results of MTT assay showed the cell toxicity can be induced in high dosages to the rAAV-*TNF* treatment group (5 × 10^5^ Gps/cell), but not the rAAV-*EGFP* transduced cells (Figure 1B). There are not difference between non-continued or continued infection groups at the doses of 5 × 10^5^ Gps/cell, thus the non-continued infection of rAAV-*TNF* (2 × 10^5^ Gps/cell, group1) was used for the subsequent combination treatment as a resistant dose. As shown in Figure 1C, the effect of MMC on rAAV-mediated TNF expression which led to cell death was depicted in different continued incubation time groups of MMC. Compared to similar treatment of rAAV-*TNF* (control), the killing effect was shown to be remarkably incubation time-dependent, and it was also found that 72 h post-incubation of MMC was critical. Moreover, the activation of caspase-3 indicated that the change of cell viability may be due to apoptosis induced when both MMC (1000 ng/mL) and rAAV-*TNF* (2 × 10^5^ Gps) were combined (Figure 1D). It was also shown here that treatment with MMC during and after AAV infection (continued treatment) is more effective than treating with MMC only during AAV infection (non-continued treatment) (Figure 1C, *p* < 0.05).

### 2.2. MMC Enhances rAAV-Mediated Transgene Expression but Does not Augment Transduction Efficiency

**Figure 2.**
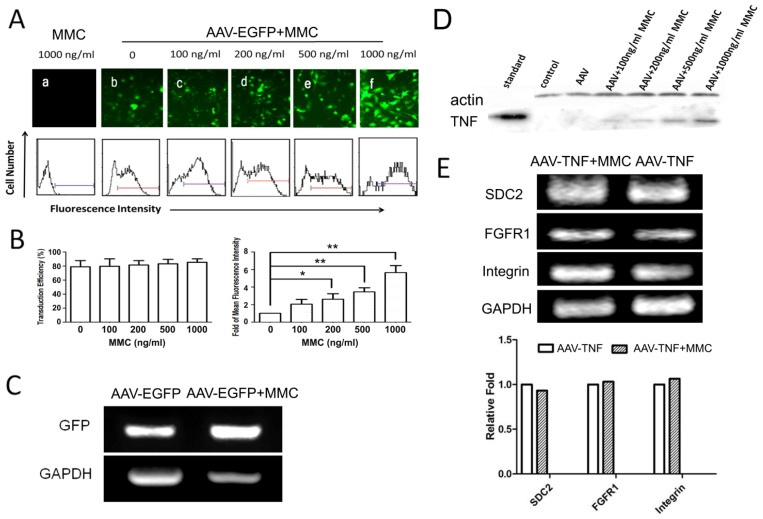
MMC enhances rAAV-mediated gene expression *in vitro*. (**A**) Fluorescence microscopy and FACS assay of U251 cells treated with MMC and AAV-*EGFP* at 72 h post-infection; (**B**) Statistical analysis for infection efficiency of combined treatment. (Left panel) transduction efficiency of AAV-*EGFP* into U251cell line at various concentration of MMC is shown. The *X* axis shows concentration of MMC, and the *Y* axis shows the percentage of positively transduced cells. The data shown are representative of three independent experiments. Values are the means ± SD (bars) of duplicate determinations; (Right panel) FACS assay for mean fluorescence intensity. The mean fluorescence intensity (MFI) of U251cells transduced by AAV-*EGFP* was measured with or without MMC treatment. Total fold change = MFI of cells treated with MMC + AAV-*EGFP*/MFI of control cells (only with AAV-*EGFP*). The data shown are representative of three independent experiments (* *p* < 0.05, ** *p* < 0.01); (**C**) RT-PCR analysis GFP mRNA expression mediated by AAV-*EGFP* or AAV-*EGFP* + MMC in U251 cells; (**D**) Western blot analysis for the effect of MMC on TNF expression mediated by rAAV-*TNF*; and (**E**) RT-PCR analysis (upper panel) and gray scale analysis (lower panel) for AAV-binding receptor mRNA expression mediated by AAV-*TNF* or AAV-*TNF* + MMC in U251 cells.

To evaluate the effect of MMC on rAAV-mediated gene expression, U251 cells infected with rAAV-*TNF* or rAAV-*EGFP* were treated with MMC at various concentrations. Both rAAV-*TNF* and rAAV-*EGFP* were used to exclude if MMC had a gene-specific effect. As indicated in Figure 2A–D, both GFP and TNF expression were dramatically increased. Furthermore, it was exhibited that enhancement effect on gene expression was dose-dependent. The mean fluorescence intensity of GFP was also found to be increased by 4 to 8 times in cells treated with MMC (200 to 1000 ng/mL) and rAAV-*EGFP* (2 × 10^5^ Gps/cell) compared with those treated with rAAV-*EGFP* (Figure 2B, right panel). In both cases, the transduction efficiency remained at the same level (about 80%, Figure 2B, left panel). GFP mRNA expression was also determined by RT-PCR, and the result was found to be consistent with that in protein level (Figure 2C).

The cell-entry step was further been investigated by the examination of the expression of rAAV receptor (SDC2) and co-receptors (FGFR1 and α5-integrin) in rAAV-permissive cell lines. Reverse transcription-PCR was used to analyze receptor expression after longtime treatment with MMC (1000 ng/mL) after rAAV-*TNF* transfection. No difference was demonstrated between MMC treated cells and control cells on mRNA level expression of receptors (Figure 2E).

### 2.3. The Effect of MMC on the Genome of Targeted Cells Infected by rAAV

**Figure 3.**
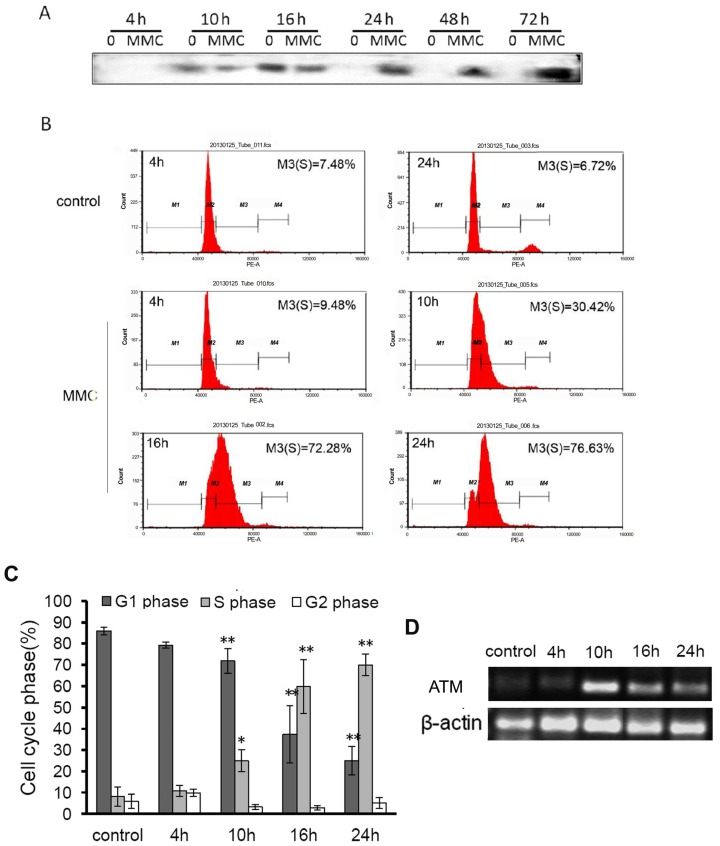
The mechanism of MMC enhancing rAAV-mediated gene expression. (**A**) the effect of MMC on rAAV’s genome by southern blot analysis; (**B**) FACS assay for the S phase arresting after MMC (1000 ng/mL) and AAV-*EGFP* (2 × 10^5^ Gps/cell) co-treatment of U251 cell, only AAV-*EGFP* treated group as control, and M1–4 represent for sub G1, G1, S and G2 phase respectively; (**C**) Statistical analysis for the effect of MMC (1000 ng/mL) and AAV-*EGFP* (2 × 10^5^ Gps/cell) co-treatment on inducing cell cycle arrested, and cell only treated with same dose of AAV-*EGFP* or MMC at 24 h as control. (* *p* < 0.05, ** *p* < 0.01); and (**D**) RT-PCR analysis for ATM mRNA expression mediated by MMC and AAV-*EGFP* (2 × 10^5^ Gps/cell) co-treatment in U251 cells.

To examine if second-strand synthesis occurred earlier in the early stage of rAAV infection and if the retention time of rAAV genome in MMC-treated cells was in the same time period as that in control cells, cell genome DNA was isolated from 4 to 72 h post-incubation after combined treatment with MMC and rAAV-*EGFP*, and was fractionated by electrophoresis on alkaline agarose gels. Southern hybridization was used to detect double-strand genome DNA in all samples. As shown in Figure 3A, genome DNA from rAAV-*EGFP* transduced cells was the same as those in MMC and rAAV-*EGFP*-treated cells in the first 16 h post-incubation, indicating that MMC and rAAV-*EGFP* co-treatment could not pace the speed of second-strand synthesis. However, DNA quantity was dramatically increased in cells after 24 h continued incubation of MMC and rAAV-*EGFP*, which meant the retention time of the rAAV genome may be extended by MMC. Interestingly, cells started to retard at S-phase during the first 16 h (57.48% ± 16.32%) after treatment with MMC and rAAV-*EGFP* (Figure 3B), and a similar effect was observed only in the MMC treated group (Figure 3C), which could also suggest that MMC played a very important role in the enhanced biological activity of rAAV during cell cycle arrest. It has also been reported that the host cell DNA repair pathway contributes to rAAV genome processing and ataxia-telangiectasia mutated (ATM) facilitates genome DNA circularization both *in vitro* and *in vivo* [22]. Thus, we detected the expression level of *ATM* at different time points after MMC treatment. It was shown that the transcriptional level of *ATM* could be increased obviously from 10 h (Figure 3D).

### 2.4. Improvement of rAAV-Mediated Gene Expression by MMC *in Vivo*

**Figure 4.**
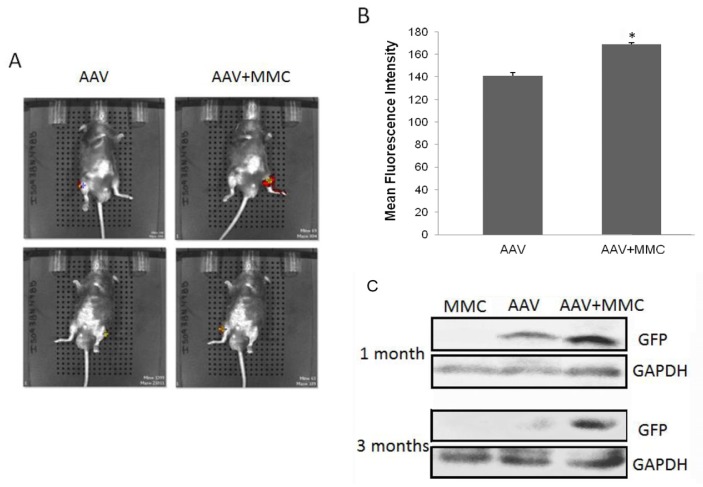
MMC improves rAAV-mediated gene expression in mice. (**A**) *In vivo* optical imaging at 1 month post-infection. (Upper panel) Right leg infected with rAAV (2 × 10^10^ Gps), left leg infected with rAAV (2 × 10^10^ Gps) mixed with MMC (1 μg/kg); (Lower panel) Left leg infected with rAAV (2 × 10^10^ Gps), right leg infected with rAAV (2 × 10^10^ Gps) mixed with MMC (1 μg/kg); (**B**) Assay of mean fluorescence intensity for *in vivo* optical imaging (* p < 0.05); and (**C**) Western blot analysis for the GFP expression mediated by rAAV with or without MMC in muscle injection model. The levels of GAPDH expression served as internal control.

Further investigation on the effect of MMC on rAAV-*EGFP* in skeletal muscle of mice administered by local intramuscular injection was also carried out. GFP expression was examined by optical imaging and western blot. As shown in Figure 4A,B, higher GFP in C57 mice was manifested in the MMC and AAV co-treated leg (whether left or right side injection), and lower GFP in the other of only AAV-treated leg. In order to test whether MMC has a long-term effect on rAAV expression, C57 mice (those who exhibited increased GFP by optical imaging at 1 month post-infection) were killed at 3 months and western blot was performed. As shown in Figure 4C, enhanced GFP expression still can be seen in MMC treated legs. However, GFP in BALB/c nude mice was not observed by optical imaging because their auto-fluorescence outweighed the GFP signal (data not shown). All of the results suggested MMC not only can improve rAAV gene expression, but also maintains this effect for a relatively long period.

### 2.5. Killing Effect of AAV-TNF with MMC after Intratumoral Injections

**Figure 5.**
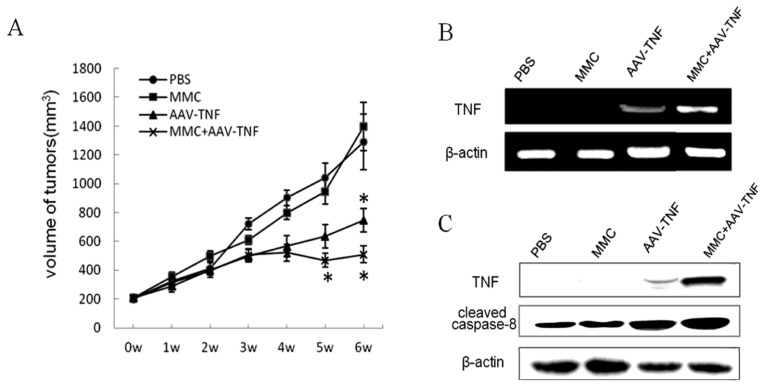
The suppression of tumor growth mediated by AAV-*TNF* combined with MMC after tumor injection. (**A**) Dynamic suppression of the tumor growth mediated by AAV-*TNF* and MMC. The mice with subcutaneous glioma xenograft were injected with AAV-*TNF* at dose of 2 × 10^10^ Gps with or without MMC (1 μg/kg), respectively. The volume of tumor was measured once a week up to 6 weeks after administration; (**B**) RNA expression of target gene in tumor tissues mediated by recombinant AAV-*TNF* combined with MMC. Reverse transcriptional PCR was performed to quantify TNF expression in tumor tissues at 6 weeks after administration of rAAV particles with MMC. The levels of β-actin RNA expression were served as internal control; and (**C**) Western blot assay for TNF protein expression and apoptosis activity induced by recombinant AAV-*TNF* combined with MMC in tumor tissues. The levels of β-actin expression served as internal control (* *p* < 0.05).

It is difficult for MMC to pass through the blood brain barrier after systemic administration, therefore local injection might be preferable for potential clinical application in glioma therapy. Thus, our main focus was on the killing effect of AAV-*TNF* with MMC after intratumoral administration. During the first 28 days of AAV-*TNF* treatment, tumor volume in all groups increased at almost the same rate. However, in the AAV-*TNF* treated group with or without MMC, tumor growth was inhibited after 35 and 42 days compared with the control groups (Figure 5A). The suppression rate of xenograft tumor growth reached up to 65% or 52% by the end of the 42nd day when the mice were killed, in AAV-*TNF* treatment groups with or without MMC, respectively, showing a strong combination inhibitory effect on tumor growth *in vivo*.

Moreover, to analyze the inhibitory effects increased by MMC treatment, the exogenous expression of TNF in implanted tumors was evaluated at the levels of transcription and translation. As shown in Figure 5B,C, significant expression of the target gene was found only in all AAV-*TNF* treatment groups, not vehicle control group, and AAV-*TNF* combined with MMC induced significant tissue or cell distribution of TNF expression. Similar results were observed from the activation of caspase-8, which can be mediated by TNF expression, in AAV-*TNF* with or without MMC treatment groups (Figure 5C). These data suggested that MMC can increase AAV-mediated TNF expression in implanted human glioma cells, which induced the obvious tumor cell killing effects.

## 3. Experimental Section

### 3.1. Cell Culture and Reagents

Human glioma cell lines (U251) was purchased from the Cell Culture Centre of Institute of Basic Medical Sciences, Chinese Academy of Medical Sciences (Beijing, China). The cells were cultured in a Modified Eagle’s medium (HyClone, #SH30024.01B; Logan, UT, USA) with 0.1 mM non-essential amino-acids (MEM-NEAA) (Gibco, #11140050; Grand Island, NY, USA). All mediums were supplemented with 10% fetal bovine serum (FBS) (HyClone, #SV30087.02; Logan, UT, USA) and penicillin-streptomycin. The cells were cultured at 37 °C in a 5% CO_2_ humidified incubator. RAAV2-*EGFP* and rAAV2-*TNF* were packaged by Benyuanzhengyang Gene Company (Beijing, China). For the construction of recombinant vectors, TNF-α cDNA was amplified by PCR and then inserted into the pSNAV2.0 expression vector (VGTC, #A20010; Beijing, China) under control of the CMV (cytomegalovirus) promoter. Recombinant AAV vector (VGTC; Beijing, China) encoding enhanced green fluorescent protein (rAAV2-*EGFP*) was constructed as control. The primary antibodies were anti-cleaved caspase-8 (Abcam, Cambridge, MA, USA), anti-TNF (Santa Cruz Biotechnology, Santa Cruz, CA, USA), and anti-β-actin (Sigma, St. Louis, MO, USA).

### 3.2. Cell Infection with rAAV and Treatment with MMC

The effects of different doses of MMC and rAAV-TNF for different infection time on cell viability were each closely monitored by MTT assay [15]. Different infection time was compared in all the experiments described below. Firstly, rAAV2-*TNF* was used to infect cells only for the first 4 h, and then the medium containing rAAV was replaced with fresh complete medium with or without rAAV, described by continued treatment group or non-continued treatment group respectively. Secondly, cells were exposed to MMC for 4 h, and the medium was replaced with the complete medium either with or without MMC, marked as continued treatment group or non-continued treatment group respectively. Finally, cells were exposed to MMC at 1000 ng/mL combined with rAAV-*TNF* of 2 × 10^5^ Gps for 4 h, and the infection medium was replaced with the complete medium either with or without MMC, marked as continued treatment group or non-continued treatment group respectively. Quantity of cell was determined by 3-(4,5-dimethylthiazol-2-yl)-2,5-diphenyltetrazolium bromide (MTT) assay at 72 h after replacing the infection media. The caspase-3/7 activation was detected at different post-infection time using Apo-One homogenous caspase-3/7 assay (Promega, Madison, WI, USA) according to the manufacturer’s instructions.

### 3.3. FACS Assay

U251 cells were removed from culture dishes and grown in 12-well plates at a density of 5 × 10^4^ cells/well. After culturing for 24 h, the medium was replaced with the FBS-free medium containing rAAV-*EGFP* at 2 × 10^5^ Gps per cell with MMC at different concentrations. After 4 h, the medium was replaced with complete medium only containing MMC. After 72 h post incubation, the expression of GFP was examined using fluorescence cell microscopy (Nikon, Kawasaki, Japan). The number of positive cells per 300 cells in three fields (100 cells/field; magnification, ×400) for each well was counted. The transduction efficiency was evaluated as percentage of positive cell numbers in total 300 transduced cells. Cells were then trypsinized, washed twice with cold PBS, and resuspended in 500 μL 1× PBS buffer for FACS analysis (Flow cytometry analysis) within 1 h with BD LSRFortessa (BD Biosciences, Franklin Lakes, NJ, USA). Ten thousand cells were counted in each analysis in this experiment. The cells only treated by different doses of MMC were used as control. At last, the mean of fluorescence intensity was assayed by BD FACSDiva Software (BD Biosciences, Franklin Lakes, NJ, USA).

The changes of U251 cell cycle mediated by MMC and AAV-*EGFP* co-treatment were analyzed by FACS assay. Firstly, U251 cells were grown in 6-well plates at a density of 2 × 10^5^ cells/well. After culturing for overnight, the medium was replaced with the FBS-free medium containing rAAV-EGFP at 2 × 10^5^ Gps per cell with MMC (1000 ng/mL), with only rAAV-EGFP treatment at the same concentration as control. After 4 h, the medium was replaced with complete medium only containing MMC. U251 cell were harvested at different times and washed with cold PBS. 70% ethanol was used to fix cells at 4 °C for overnight. The cells were re-suspended with 5 mL cold PBS and stained by PI staining buffer. After two times wash, about 1 × 10^5^ cells were used for flow cytometry (BECKMAN Cytomics FC 500, Beckman-Coulter, Fullerton, CA, USA), and PI red fluorescence channel was detected and analyzed.

### 3.4. Western Blot Assay

Cells treated in the same way were lysed at 72 h post incubation, and total proteins were collected and separated in SDS-PAGE (sodium dodecyl sulfate polyacrylamide gel electrophoresis) for further immunological assay. The primary rabbit anti-TNF antibody, mouse anti-actin antibody and IgG secondary antibodies labeled with horseradish peroxidase (HRP) were used to detect positive signals. ECL reagent (Millipore, #WBKLS0100, Bedford, MA, USA) was followed by X-ray film developing.

### 3.5. RT-PCR Assay

The target gene expression in mRNA level was measured by reverse-transcription PCR. Total RNA was extracted using TRIzol^®^ Reagent (Invitrogen, #15596; Carlsbad, CA, USA). Specific primers were derived from the coding region of the genes: GFP-F: 5′-CAGAAGAACGGCATCAAG-3′, GFP-R: 5′-GGGGTGT TCTGCTGGTAG-3′; TNF-F: 5′-GAAAGCATGATCCGGGACGTGGA-3′, TNF-R: 5′-GTTGG ATGTTCGTCCTCCTCACA-3′; ATM-F: 5′-GCTCTTCA GGTCTAAATCA-3′, ATM-R: 5′-TCTCCTAATTCACACACTC-3′; SDC2-F: 5′-CTGATGAGGATGTAGAGAGT-3′, SDC2-R: 5′-TTGTCTGAGCAGGTATCT TG-3′; FGFR1-F: 5′-TGGAGTTCATGTGTAAGG-3′, FGFR1-R: 5′-TCAAGA TCTGGACATAAGG-3′; α5-Integrin-F: 5′-AGACATTGGCACCTAATC-3′, α5-Integrin-R: 5′-ATTCCTGGCTTCTCCTAA-3′; GAPDH was used as control: GAPDH-F: 5′-CCTGCT TCACCACCTTCTTG-3′; GAPDH-R: 5′-CCATCACCATCTTCCA GGAG-3″. Synthesis of the first strand cDNA was performed at 42 °C in a period of 1 h. PCR were performed with 30 cycles and the products were fractionated on a 2% agarose gel. All primers were synthesized by Sangon Biotech Co. Ltd. (Shanghai, China).

### 3.6. Southern Blot Assay

Southern blot was used to investigate the second-strand synthesis of rAAV-EGFP in the nucleus at different post infection time either in the presence or absence of MMC. Cells were collected and lysed at a post-infection time interval of 4, 10, 16, 24, 48, 72 h, respectively. Genomic DNA was isolated and digested overnight with Xho I (NEB, #R0146V; Beverly, MA, USA), and loaded onto a 1.5% agarose gel. After denaturation and neutralization, the DNA was transferred onto a nylon filter membrane for pre-hybridization and hybridization. Biotin DecaLabel™ DNA Labeling Kit (Fermentas, #K0651; Hanover, MD, USA) was used to generate and label DNA probes of EGFP gene according to manufacturer’s instructions. The blots were washed and detected as described in the instructions of Biotin Chromogenic Detection Kit (Fermentas, #K0661; Burlington, ON, Canada).

### 3.7. Experimental Animal Model and Tumorigenic Assay

C57 mice (*n* = 6) of 6 to 8 weeks of age (about 20 g) were used in this study. Each of C57 mouse was injected with rAAV-EGFP (2 × 10^10^ Gps) mixed with physiological saline (PBS) into one leg as control, while rAAV-EGFP with MMC (1 μg/kg) into the other leg. *In vivo* optical imaging was used to detect the GFP expression at 1, 3, 7, 14 and 30 d, respectively, after injection. Skeletal muscles were sampled at post-infection period of 1 and 3 months and proteins were extracted followed by western blot as mentioned before. All experiments were repeated three times.

To establish human glioma xenografts animal model, 5 × 10^6^ U251 cells were subcutaneously inoculated into dorsal flanks of 4–6 weeks old male Balb/c nude mice (*n* = 6). Intratumor injection of the recombinant virus (2 × 10^10^ Gps) with or without MMC (1 μg/kg) was performed when the tumors reached 200 mm^3^, and MMC (1 μg/kg) or vehicle (1× PBS) at same volume was used as control. Tumor size was measured at least once a week with a caliper for two-dimensional longest axis (*a*) and shortest axis (*b*). The tumor volume was calculated using the following formula: *V*(mm^3^) = (*ab*^2^)/2. The animals were sacrificed 6 weeks after administration of the recombinant virus and the removed tumors were weighted and used for Western blot assay or tissue sections. All experiments were repeated three times.

### 3.8. Statistical Analysis

Results of the data are reported as the mean ± standard error (SD) for three independent experiments. Data evaluating different conditions were compared using the *T*-test for paired samples or ANOVA (analysis of variance), and the significant level was defined as *p* < 0.05.

## 4. Conclusions

RAAV with its superior ability of biological infection, low immunogenicity and non-pathogenicity have rapidly gained in popularity for gene therapy in the past decades. A number of first-phase clinical trials using rAAV gene delivery systems have been completed. However, low transgene expression leading to high dosage and safety problems in clinical trials has been problematic in AAV application. On the other hand, increasing rAAV biological activity by DNA damaging agents such as antitumor drugs and radiation has been reported. Therefore, the combination of AAV gene therapy with chemotherapy or radiotherapy is being considered as a promising method in cancer treatment.

MMC is widely used as an antitumor drug due to its activity of crosslinking DNA. In this study, it is demonstrated that MMC can enhance rAAV-mediated transgene expression in AAV-permissive cell lines without increasing the transduction efficiency, because there is no difference in percentage of positive cell numbers in a total of 300 transduced cells with or without MMC treatment (Figure 2B). Moreover, the related receptors’ expression of rAAV transduction has no difference between MMC treated cells and normal cells. These findings suggested that improvement of rAAV biological activity by MMC is independent of AAV receptor and co-receptors’ mediated endocytosis step, when rAAV was taken up by the target cells, but may be related to the downstream transduction pathways.

Second strand synthesis is a rate determining step in AAV transduction. Several studies have reported that AAV-mediated transgene expression by facilitating AAV genome conversion can be enhanced by DNA damage agents or radiation [12,13,19,23]. In this study, double-strand genome DNA over time has been monitored, but no increased conversion efficiency was observed before 24 h post-incubation. Instead, AAV genome stability was increased due to MMC after 24 h and longer retention time was exhibited. Sanlioglu *et al*. reported that the enhancement of rAAV transduction by UV was due to the increased abundance of circular rAAV genome [24]. Previous studies also confirmed that ATM/ATR activated by double strand breaks (DSBs) could activate downstream kinases leading to intra-S checkpoint followed by DNA replication inhibition [25–27] reported that MMC treatment which led to formation of DSBs in chromosomal DNA activated S-phase checkpoint pathway. Walsh *et al*. [28] also indicated that MMC co-treatment can show DNA correction in the hemotopoietic cells after AAV transduction. Interestingly, in our study, retardation was observed during the cell cycle at S-phase in MMC treated cells after 10 h (Figure 3B), meanwhile, the mRNA expression of ATM could be increased from 10 h (Figure 3C). Therefore, MMC may induce cell cycle retardation and affect ATM, which might lead to increased rAAV genome circularization followed by genome stability. Thus, this relationship of ATM and improvement of rAAV by MMC should be given further study.

We observed enhanced gene expression induced by MMC at 42 days in xenografts of human glioma tumor implants and at one month and three months in intramuscular model *in vivo*, that suggested that the rAAV genome remained stable in the long term. The metabolization of MMC is rapid *in vivo* and it will not last for three months. Thus, it suspected that facilitated rAAV genome circularization in the first few days was caused by the longer retention time induced by S-phase retardation and consequently contributed to the higher expression over several months. However, further investigation is needed to understand how the rAAV genome is affected by MMC and the fate of the rAAV genome.

Finally, in the animal experiments, only 2 × 10^10^ Gps recombinant virus followed with 1 μg/kg MMC per mouse were injected, levels lower than the normal therapeutic dose, but the effect on improving target gene expression and tumor cell killing was still demonstrated. Our findings thus conclude that rAAV-mediated cancer gene therapy might be used as the predominant therapy in the future, with chemotherapy applied in conjunction.
